# Temperature differences between sites lead to altered phenylpropanoid metabolism in a varietal dependent manner

**DOI:** 10.3389/fpls.2023.1239852

**Published:** 2023-10-19

**Authors:** Kelem Gashu, Pankaj Kumar Verma, Tania Acuña, Nurit Agam, Amnon Bustan, Aaron Fait

**Affiliations:** ^1^ The Albert Katz International School for Desert Studies, The Jacob Blaustein Institutes for Desert Research, Ben-Gurion University of the Negev, Be'ersheba, Israel; ^2^ Wyler Department of Dryland Agriculture, French Associates Institute for Agriculture and Biotechnology of Dryland, The Jacob Blaustein Institutes for Desert Research, Ben-Gurion University of the Negev, Be'ersheba, Israel; ^3^ Ramat Negev Desert Agro-Research Center, Ramat Negev Works Ltd., Hazula, Israel; ^4^ Albert Katz Department of Dryland Biotechnologies, French Associates Institute for Agriculture and Biotechnology of Dryland, The Jacob Blaustein Institutes for Desert Research, Ben-Gurion University of the Negev, Be'ersheba, Israel

**Keywords:** phenylpropanoid metabolism, elevated temperature, *V. vinifera*, climate change, LC-MS, plasticity

## Abstract

Elevated temperature has already caused a significant loss of wine growing areas and resulted in inferior fruit quality, particularly in arid and semi-arid regions. The existence of broad genetic diversity in *V. vinifera* is key in adapting viticulture to climate change; however, a lack of understanding on the variability in berry metabolic response to climate change remains a major challenge to build *ad-hoc* strategies for quality fruit production. In the present study, we examined the impact of a consistent temperature difference between two vineyards on polyphenol metabolism in the berries of 20 red *V. vinifera* cultivars across three consecutive seasons (2017–2019). The results emphasize a varietal specific response in the content of several phenylpropanoid metabolites; the interaction factor between the variety and the vineyard location was also found significant. Higher seasonal temperatures were coupled with lower flavonol and anthocyanin contents, but such reductions were not related with the level of expression of phenylpropanoid related genes. Hierarchical clustering analyses of the metabolic data revealed varieties with a location specific response, exceptional among them was Tempranillo, suggesting a greater susceptibility to temperature of this cultivar. In conclusion, our results indicate that the extensive genetic capacity of *V. vinifera* bears a significant potential to withstand temperature increase associated with climate change.

## Introduction

Climate models predict an increase in the atmospheric temperature in many areas where viticulture currently dominates agricultural production (Santos et al., 2020). This may result in decline in grape production and quality in the affected areas (Morales-Castilla et al., 2020). In Mediterranean-like climates, a significant advancement of the grapevine phenological phases due to increasing temperatures, shifts berry ripening to the warmest part of the season (Venios et al., 2020); berry dehydration and shriveling can follow. Red wine grape cultivars appear to be more sensitive to climate shifts than white cultivars (Gashu et al., 2020), due to the longer maturation period required by red varieties exposing the berries to longer periods of heat. A large variability in phenological sensitivity between cultivars was found in our earlier study (Gashu et al., 2020).

Grape berry metabolism strongly depends on environmental factors (Rienth et al., 2021), which can modulate major biochemical pathways, including primary carbon and polyphenol metabolism, and lead to altered crop qualities. Polyphenol compounds such as anthocyanin and flavonols are part of an array of metabolites imparting protection to the fruits from environmental hazards, including temperature and radiation (Weisshaar and Jenkins, 1998; Winkel-Shirley, 2002). However, extreme environmental conditions such as high temperature and strong solar radiation can lead to their degradation (Reshef et al., 2018; Reshef et al., 2019). For instance, berry shriveling and low fruit anthocyanin and flavonol accumulation are typical attributes of grapes grown in warm climates (Šuklje et al., 2016; Martínez-Lüscher et al., 2020; Deloire et al., 2021), the severity of which depends on the variety. Severe heat stress (>35 °C) can lead to a decreased or arrest of sugar accumulation as well as inhibition of berry growth, resulting in a delay of ripening (Lecourieux et al., 2014) due to a lack of carbon supply by photosynthesis (Keller, 2010). A more recent study on Cabernet Sauvignon and Sauvignon Blanc grapes shows that even a moderate increase in temperature (~1.5°C increase in bunch zone air temperature) reduces polyphenols and aroma quality of grape berries (Wu et al., 2019). Elevated temperatures were shown to repress major anthocyanin biosynthesis regulators such as *VviMYBA1* and downstream genes such as *VviUFGT, VviCHI, VviF3H2, VviDFR, and VviLDOX* (Pastore et al., 2017; Yan et al., 2020; Rienth et al., 2021). 

While most of the studies investigating temperature effects on skin polyphenol focused on a single or very few cultivars (Greer and Weedon, 2013; Gouot et al., 2019; Wu et al., 2019; Ryu et al., 2020), its broader effect remains poorly understood. The present study employs a varietal collection of twenty red wine grapevine cultivars, grown in two locations differing by 2°C average temperature to further testing the hypothesis that the broad genetic diversity among *V. vinifera* bears the key to high performances in warm climates (Wolkovich et al., 2018)

Previous investigation on the same collection assessed the diversity existing in phenology and central metabolism among cultivars and indicated that the warmer site led to extended fruit ripening phases, causing berry shriveling and cluster collapse in few red cultivars such as Pinot Noir, Ruby Cabernet, and Tempranillo (Gashu et al., 2020). Here we studied the inter-varietal response in berry polyphenol metabolism and regulation in response to a multi-seasonal temperature shift.

## Materials and methods

### Experimental layout

**Figure 1:**
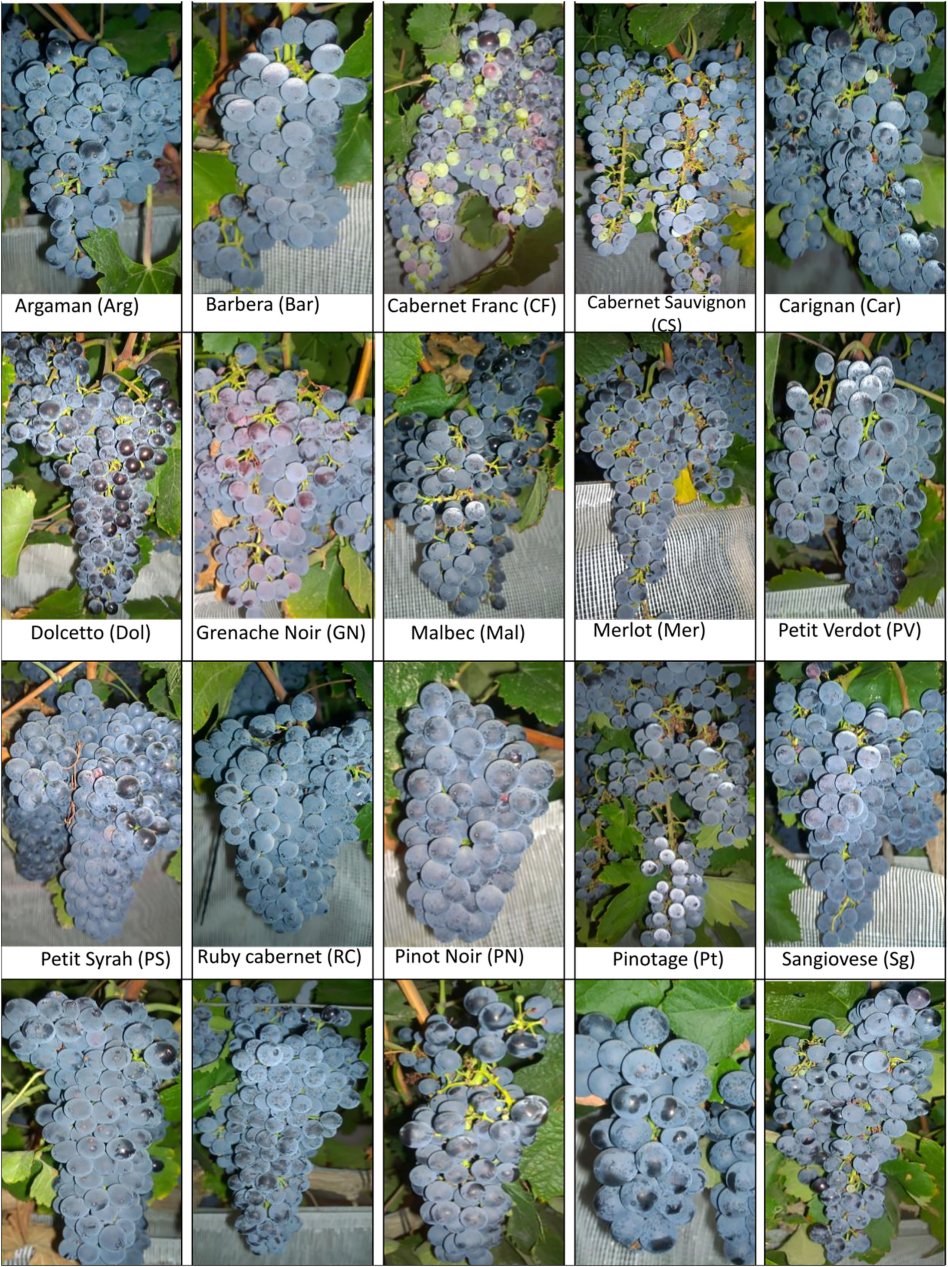
List of cultivars used in the experiment. Cultivar names are composed by abbreviations in the bracket. The pictures were taken by the Fait Lab research team in 2017. The background mesh was used as a protective measure to prevent birds from damaging the berries.

The experiments were conducted over three consecutive seasons, from 2017 to 2019, in two vineyards located in the Negev Highlands in Israel: the Mitzpe Ramon (MR) vineyard (30°38’48.6”N 34°47’24.5”E, 850 m asl) and the Ramat Negev (RN) vineyard at the Desert Agro-Research Center (30°58’43.4”N 34°42’31.6”E, 300 m asl). Both vineyards shared the same experimental setup, comprising 20 red wine cultivars (**Figure 1**), grafted onto 140 RU rootstock; both vineyards were planted in 2012 in a randomized block design, as described earlier (Gashu et al., 2020). The RN vineyard experienced a slightly higher daily mean temperature (with a difference of 1.5°C) and vapor pressure deficit (VPD) compared to MR over the course of the three seasons; **Supplementary Figure 1** shows the differences in VPD between locations over the three seasons. The climatic conditions between the two vineyards have been extensively described in Gashu et al. (2020).

Daily degree days (DDD) were calculated following the method described by Jones et al. (2010); the sum of DDD from *véraison* to harvest was computed for each cultivar to assess the DDD effect on fruit quality parameters, as follows:


(1) $$\text{DDD}=\;\sum_{\text{H}arvest}^{\text{V}eraison}\text{max}\left[\left(\frac{\left[\text{T}max+Tmin\right]}{2}\right)-10,0\right]$$

Where, *Tmax* is the daily maximum temperature and *Tmin* is the daily minimum temperature.

To evaluate the effect of maximum and minimum temperatures on fruit metabolism, we introduced accumulated degree hours indices expressing heat stress (Hs) and relaxation (Relx). Hs and Relx count, for each cultivar, the duration from *véraison* to harvest during which the hourly air temperatures were higher than 30°C and lower than 20°C, respectively.


(2) $$Hs=\Sigma_{Harvest\;}^{Veraison}\;max\left[\left(Temp-30\right),0\right]$$

(3) $$Relx=\Sigma_{Harvest\;}^{Veraison}\;max\left[\left(20-Temp\right),0\right]$$

Where, *Temp* is hourly temperature.

### Berry sampling

During each season, at *véraison* and harvest, berries were sampled for metabolite extraction and berry indices. At *véraison*, each cultivar was sampled when berries reached approximately 50% color change. At harvest, berries were sampled from each cultivar approaching the 23 ± 1°Brix level. Samples were collected in the morning from four biological replicates at each location from each cultivar. In each sampling, at least 30 berries per replicate were pooled from five different vines in each block on the east side of the vine (six berries per vine were sampled from the top, middle, and bottom of the bunch), and immediately snap-frozen in liquid nitrogen. Berries were peeled while still frozen, by carefully separating the skin from the pulp, and the seeds were removed. The skin was kept at -80°C until further analysis.

### Berry skin extract preparation

Grape skin samples were lyophilized and ground under liquid nitrogen using a retsch‐mill (Retsch, Haan, Germany) with pre-chilled holders and grinding beads. For metabolite extraction, 40 mg of frozen skin powder was weighed and extracted in a 1-ml pre-cooled methanol:chloroform:water extraction solution (2.5:1:1 v/v) with ampicillin (1 mg ml^−1^ in water) and corticosterone (1 mg ml^−1^ in methanol) as internal standards as described by Hochberg et al. (2013) and Degu et al. (2014). Skin extracts were filtered (0.22 μm Millipore, MA, USA) and transferred to glass vials for analysis using ultra-performance liquid chromatography coupled to a quadrupole time‐of‐flight mass spectrometer (UPLC QTOF‐MS; Waters, MA, USA).

Chromatographic separation and the MS conditions were exactly as described previously (Hochberg et al., 2013).

### LC-MS data processing and annotation

MassLynxTM version 4.1 (Waters) was used for system control and data acquisition. Metabolites were annotated based on fragmentation patterns searched against those in the Chemspider metabolite database (http://www.chemspider.com/), and the consistency of their retention times with those of identified metabolites was compared with the data in the scientific literature.

### RNA extraction and gene expression analysis

Frozen ground skin tissue from the same sample used for metabolite extraction were used for gene expression analysis as previously described by Degu et al. (2014). RNA extraction was performed from three biological replicates of 50 mg of berry skin tissues sampled at *véraison* and harvest in the above mentioned five cultivars, grown at RN and MR in 2019 season. Given that the climate conditions in 2019 resemble the typical Negev desert climate, samples collected during the 2019 season were used for RNA extraction. Total RNA from grape skin tissues was extracted using *Quick*-RNA™ Miniprep Kit (https://www.zymoresearch.com), following the manufacturer’s instructions, with minor modifications. Briefly, RNA was extracted using buffer containing CTAB (2%), 150 mM trisodium citrate, 20 mM Na_2_EDTA, 6M guanidine hydrochloride, PVP40 (2%) and 1% β-ME The extracted RNA was subsequently precipitated using isopropanol and then subjected to column purification. The cDNA was synthesized using a qScript™ cDNA Synthesis Kit (https://www.quantabio.com) using 250 ng of purified RNA following the instruction manual. The qPCR was performed with an ABI 7500 Real-Time PCR system (https://www.thermofisher.com) using Power SYBR^®^ Green (https://www.thermofisher.com) and data acquisition was done by 7500 software v2.3 (Applied Biosystems, USA). All reactions were performed in triplicates from three sets of biological replicates. Relative quantification was performed using the Actin gene as an internal reference gene, and RNV as a calibrator sample by the 2^-ΔΔCT^ method (Livak and Schmittgen, 2001).

### Statistical analysis

All the statistical analyses were performed using ‘R’ version 3.6.0 (R Core Team, 2017). A three-way factorial analysis was used to assess the effects of cultivar (C), location (L), and growing season (Y), and the interactions between them, using the built-in *aov* function. The differences between locations for each cultivar were tested using the *Wilcox.test* function. Clustered heatmaps were created using *Complexheatmap* (Gu et al., 2016). Clustering of samples was calculated by Euclidean distances and the Ward.D2 clustering method in the functions *get_dist* and *hclust*, and the built‐in “dendextend” and “factoextra” packages (Galili, 2015). Correlation-based network analyses were conducted using the MetScape application and the NetworkAnalyzer tool, available in Cytoscape version 3.7.2. All correlation analyses and preparation for network visualizations were generated in ‘R’ using the built‐in cor function with the “Pearson” algorithm. Correlations were incorporated into the network if they were statistically significant (*p* value< 0.05) and their correlation coefficient was higher than 0.3 or lower than −0.3. Principal component analysis (PCA) was plotted using JMP^®^ version 13 (SAS Institute Inc., Cary, NC, 1989–2007). The norm of reactions that represents phenotypic changes (y-axis) due to location and seasonal climate changes (x-axis), was calculated using linear regression models for each trait computing the slope. The slope represents phenotypic plasticity.

## Results

### Phenylpropanoid diversity among cultivars of *Vitis vinifera* as affected by season and location

Metabolite profiles obtained from berries, sampled at *véraison* during the 2017, 2018 and 2019 seasons, showed distinct patterns between cultivars and seasons (**Supplementary Figure 2**). At *véraison*, HCL analysis highlighted the separation between seasons (**Supplementary Figure 2**). Inter-seasonal differences were likely attributed to a lower anthocyanin content in 2017 samples. In contrast, 2018 samples grouped among 2017 and 2019 clusters showing an in-between pattern of change in metabolite content. Within each seasonal cluster, cultivars were grouped by location. Among the cultivars, only Sangiovese, Pinot noir and Grenache noir, which undergo *véraison* earlier than others, did not separate neither by season nor by location (**Supplementary Figure 2**).

**Figure 2:**
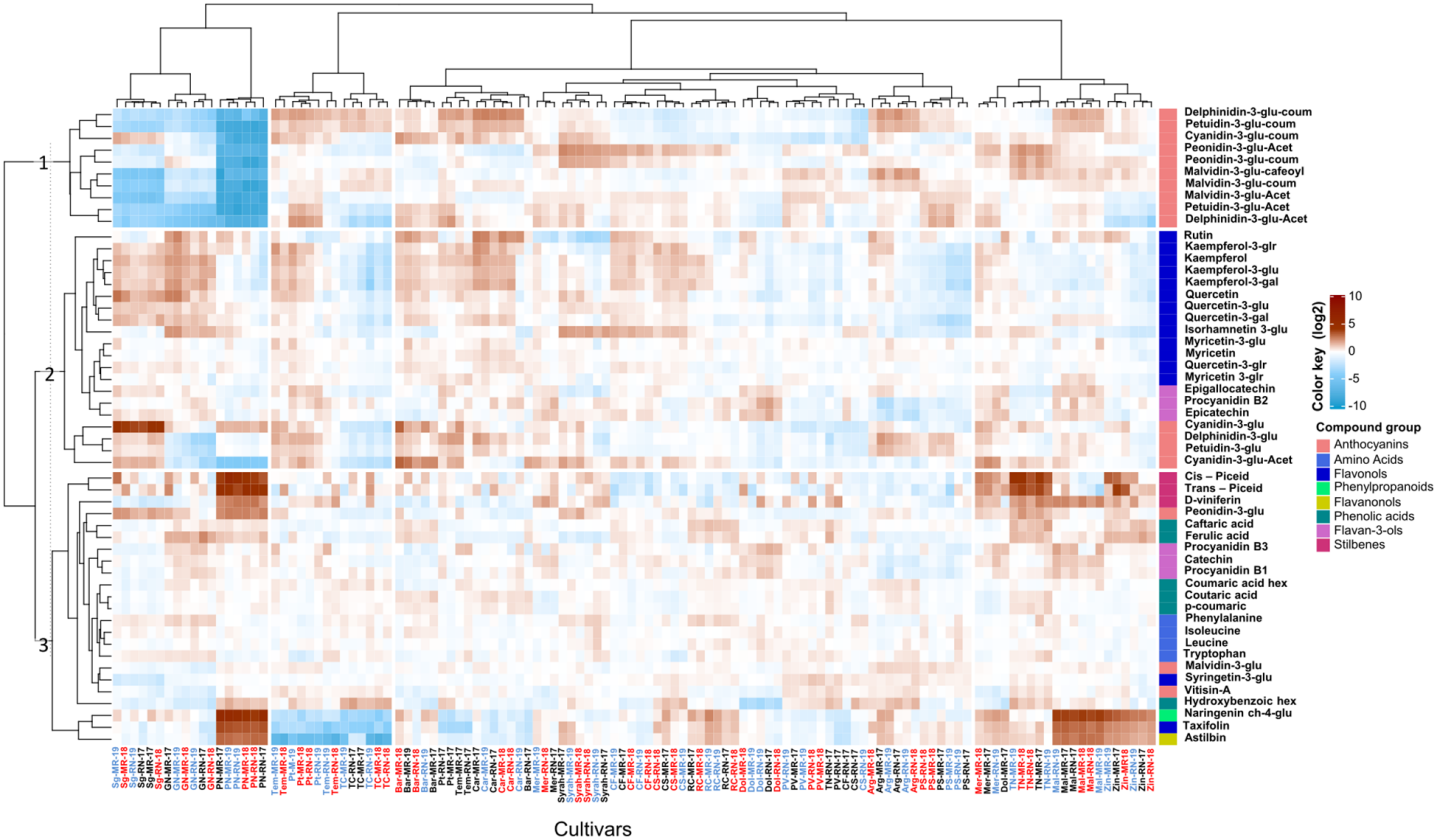
Heatmap of skin polyphenols in red grapevine berries at harvest. The heatmap was generated using the mean value of four biological replicates following normalization to the median of each metabolite on all cultivars and log2 transformation. Cultivar names are denoted by cultivar abbreviations, vineyard abbreviations (MR and RN) followed by vintage (17,18 or 19). Colored cultivar names indicate samples collected in 2017 (black), 2018 (red), and 2019 (blue). Red and blue rectangles represent an increase and decrease of metabolite relative to the median. MR, Mitzpe Ramon; RN, Ramat Negev.

At harvest, considerable varietal differences were evident compared to *véraison* regardless of the vineyard location (**Figure 2**). Through HCL analysis, we identified metabolites that exhibit a predominant cultivar effect. The lowest anthocyanin content was shown in Pinot noir, Sangiovese, and Grenache Noir (**Figure 2**; **Supplementary Table 1**) irrespective of the season. Exceptions were cyanidin-3-glucoside (157.66 and 142.45 µg g^-1^ DW at MR and RN, respectively) highest in Sangiovese, and peonidin-3-glucoside (3.64 and 4.84 µg g DW^-1^ at MR and RN, respectively) in Pinot noir (**Figure 2**; **Supplementary Table 1**). Among the stilbenes, higher content was measured in Pinot noir and Touriga nacional cultivars; where the relative content of *trans* piceid and *cis* piceid were 10-fold higher than in Cabernet Franc and Cabernet Sauvignon. Regardless of the location, Pinot noir, Malbec, and Zinfandel exhibited relatively high levels of naringenin chalcone-4-glucoside, taxifolin and astilbin. In contrast, the lowest content of these metabolites was observed in Pinotage, Tempranillo and Tinta cao cultivars (**Figure 2**).

**Figure 3:**
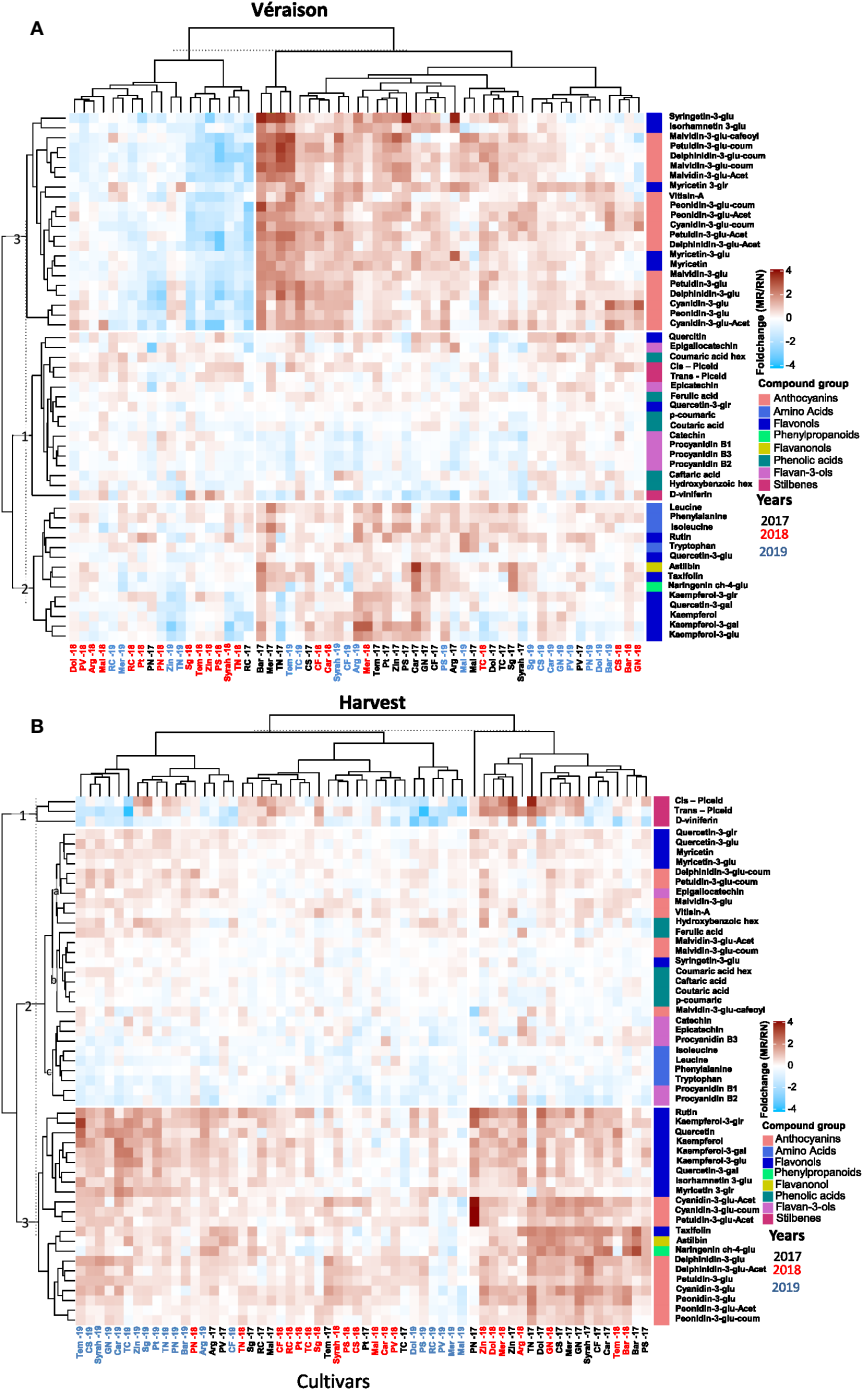
The variability in skin polyphenols of red grapevine berries at *véraison* **(A)** and harvest **(B)** between the location during 2017-2019, expressed as the foldchange of average response values (Mitzpe Ramon (MR)/Ramat Negev (RN)). The mean value of four biological replicates of each cultivar in each location and season was calculated separately. Then, the MR values were divided by the RN values and transformed to log2. The hierarchical clustering heatmap was generated using the log2-transformed data. Cultivar names are composed by vintage abbreviations (17, 18, 19). Colored cultivar names indicate samples collected in 2017 (black), 2018 (red), and 2019 (blue). Colored rectangles represent metabolite increases at MR (red) and RN (blue). A mirror heat map of significance values is presented in **Supplementary Figure 3**.

When values were expressed as fold-change to visualize the differences in metabolite content between locations (**Figure 3**), three groups of metabolites were of particular interest at *véraison*. Cluster (1) comprised of metabolites showing minor differences between locations regardless of the seasons, e.g. phenolic acids, flavan-3-ols, stilbenes, and quercetin and quercetin-3-glucuronide, among flavonols (**Figure 3A**; **Supplementary Figure 3**); cluster (2) included mainly flavonols, flavanonols, and aromatic amino acids such as tryptophan and phenylalanine, measured at higher content in MR samples, particularly in the 2017 season while differences between the sites were cultivar dependent in the 2018 and 2019 seasons; cluster (3) was composed of anthocyanins, myricetin and its aglycons, syringetin-3-glucoside and isorhamnetin-3-glucoside, that markedly accumulated in MR during the study with the exception of season 2018 (**Figure 3A**), when high spring temperature likely triggered the biosynthesis of anthocyanins in *véraison* berries at the warmer RN.

At harvest, cluster (1) included metabolites displaying inter-seasonal variability between the vineyard sites, e.g. stilbenes (*trans* piceid, *cis* piceid and D-viniferin) markedly accumulated at MR in the 2017 and 2018 seasons but not in 2019; cluster (2) included metabolites displaying minor differences between locations, e.g. phenolic acids, amino acids, flavan-3-ols, a few coumaroylated anthocyanins, and quercetin and myricetin aglycones; and cluster (3) was characterized by metabolites showing predominant location effect (**Figure 3B**), mainly flavonols and anthocyanins, generally lower at warmer RN with very few exceptions among cultivars, such as Ruby Cabernet, Merlot, Malbec, and Petit Verdot.

### The pace of metabolites change from *véraison* to harvest was higher at RN than MR

For the purpose of visualizing the pace of metabolite pattern of change from *véraison* to harvest in each location, phenylpropanoid profiles obtained from berries at harvest were divided to the values at *véraison* (harvest/*véraison*), and metabolite response values were log transformed prior to HCL analysis. Three clusters were of particular interest at both locations (**Supplementary Figures 4A, B**). Cluster (1) included metabolites which decreased towards harvest, and included flavan-3-ols, phenolic acid, quercetin and its aglycons, rutin, kaempferol-3-glucuronide, taxifolin, naringenin ch-4-glu and astilbin. The extent of decrease in metabolite content from *véraison* to harvest was greater at warmer RN, where seasonal fluctuation (**Supplementary Figure 4**, cluster 1) were also observed. In contrast, at MR the extent of decrease remained consistent across the three seasons. Cluster (2) and cluster (3), comprising mainly of flavonols, amino acids, stilbenes, and anthocyanins, which generally increased during berry ripening at both sites.

### Network analysis reveals location-specific metabolite-to-metabolite and metabolite-to-environmental factor correlations

**Figure 4:**
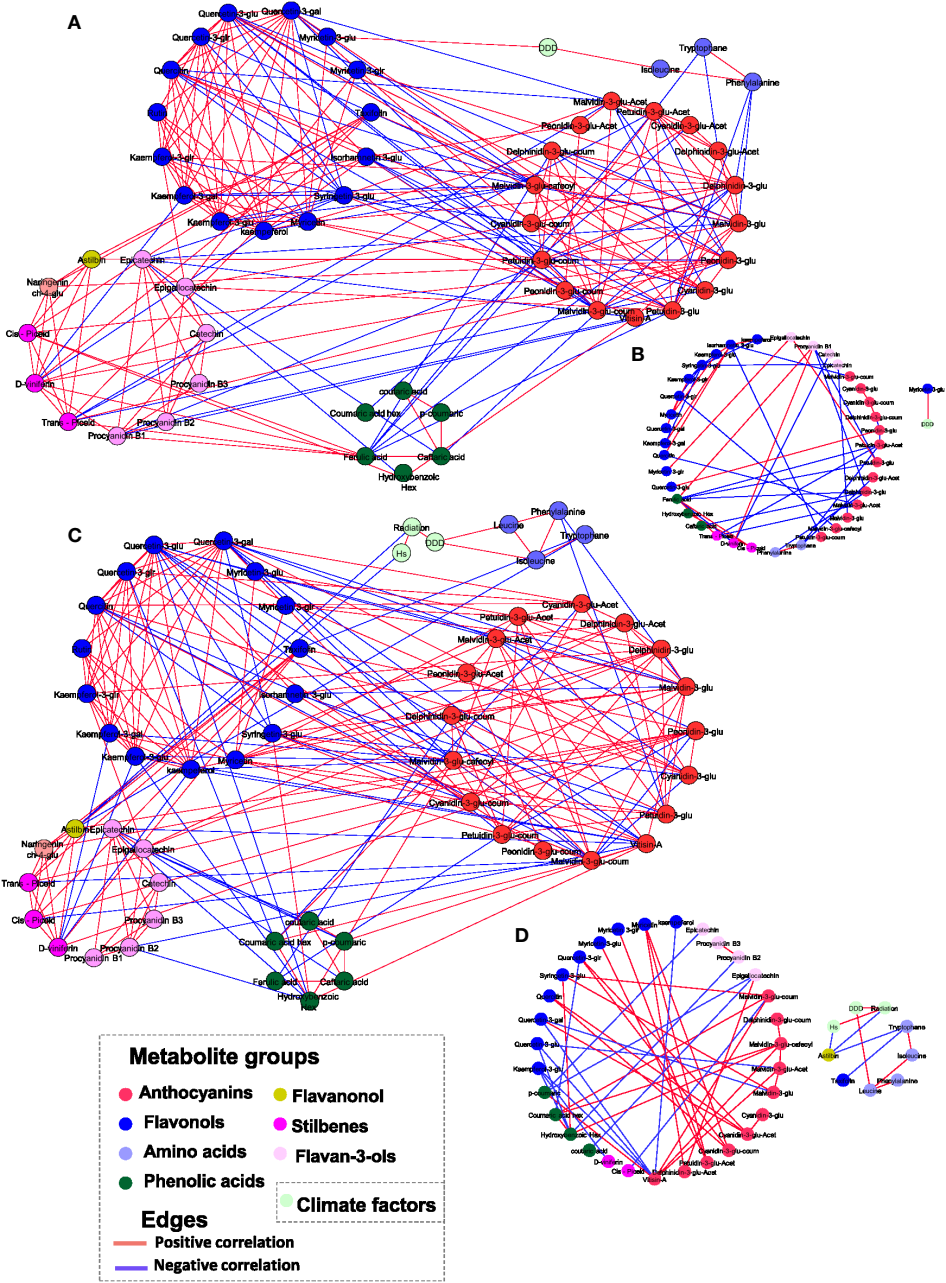
Correlation-based metabolite networks at harvest. Network visualization of skin polyphenols and environmental factors correlated to each other as measured in 20 red grapevine cultivars grown in Mitzpe Ramon **(A)** and Ramat Negev **(C)** in 2017, 2018 and 2019. Metabolites are color-coded according to the chemical groups. The correlation analysis was performed using four biological replicates at Mitzpe Ramon and Ramat Negev vineyards in each season, and only significant correlations are presented. Positive correlations are shown as red edges, and negative correlations as blue edges. Correlations were based on Pearson’s method. **(B)** Network unique to Mitzpe Ramon samples. **(D)** Network unique to Ramat Negev samples.

Differences between the vineyards in respect to the relations between metabolites and climate factors (such as DDD, Hs, and Relx) were explored by correlation-based network analysis. First, we constructed networks for RN and MR samples separately (**Figures 4A, C**). The correlation threshold level for all networks was set at *p*-value< 0.05 and ‘*r* > 0.4 or *r*< −0.4’. The results showed that the RN network was characterized by a slightly higher number of edges (222) and nodes (56) than MR (216 and 53 edges and nodes, respectively) (**Supplementary Table 2**).

When networks were constructed containing edges and nodes unique to each site (i.e. differential graphs), results showed a similar number of edges at both sites (**Figures 4B, D**). However, the relation between anthocyanin and flavonols was different between the locations. In the MR network, glycosylated, acylated, and p-coumaroylated anthocyanins were negatively correlated with flavonols (quercetin, quercetin-3-glucuronide, and isorhamnetin-3-glucoside) and phenolic acids such as ferulic and caftaric acids (**Figure 4B**). In contrast, in the RN network, anthocyanins were positively correlated with flavonols, with the exception of the negative correlation of quercetin-3-O-glucoside and quercetin-3-glucuronide with Malvindin‐3‐O‐(6″‐acetyl‐glucoside) and malvidin‐3‐O‐glucoside, respectively (**Figure 4D**). In both networks, only a few metabolites were linked to environmental factors. In the MR network, only myricetin-3-O-glucoside was positively correlated with DDD, while none of the metabolites were correlated with Hs and Relx. In the RN network, leucine was positively correlated with DDD, whereas astilbin was negatively correlated with Hs and accumulated radiation. It should be noted that the network analysis was performed combining the data of all cultivars, which may conceal the response of individual cultivars to environmental elements.

### Hierarchical clustering and correlation analyses revealed Tempranillo as the most climate sensitive cultivar

**Figure 5:**
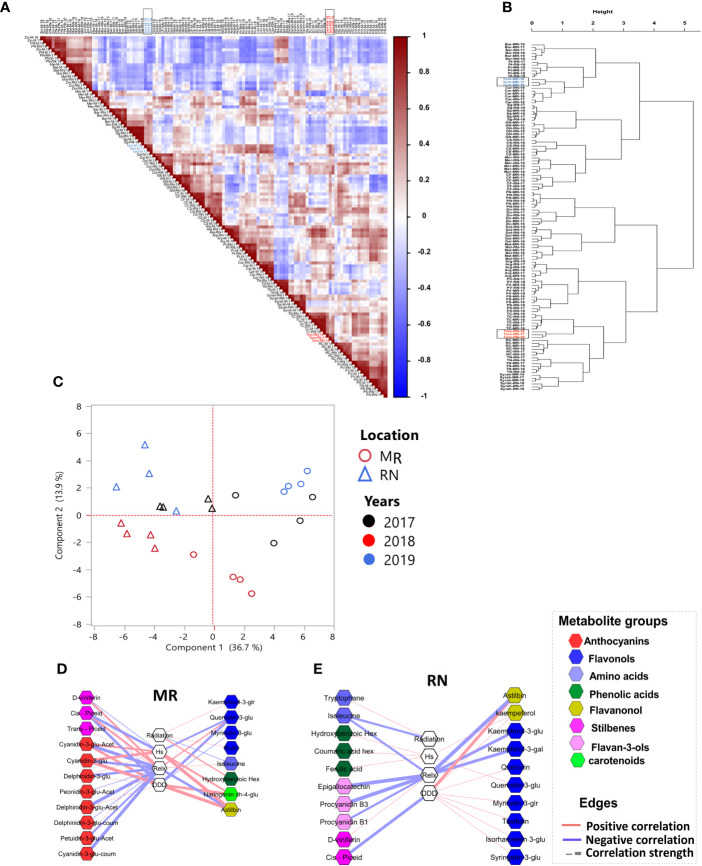
Unsupervised analysis of the skin metabolic plasticity of red berries at harvest. **(A)**; Pearson’s correlation analysis and **(B)**; hierarchical clustering based on Pearson’s correlation distance matrix show Tempranillo berries (marked in a black rectangle) separated based on location. **(C)**; principal component analysis of skin metabolites in Tempranillo berries, **(D, E)** network visualization of correlations between skin metabolites and climatic factors, calculated from *véraison* to harvest, in Tempranillo grape. Metabolites are color-coded according to the chemical groups. The correlation analysis was performed using four biological replicates at Mitzpe Ramon (MR) and Ramat Negev (RN) vineyards in each season, and only significant correlations are presented. Correlations were based on Pearson’s method. Cultivar names are composed by location abbreviation (MR or RN) and vintage (17, 18, 19). MR; Mitzpe Ramon, RN; Ramat Negev, Hs; accumulated heat stress hours, Relx; Accumulated relaxation hours, DDD; accumulated daily degree days.

To compare skin polyphenol metabolites in the berries, a Pearson’s correlation matrix was created using UPLC-QTOF-MS generated metabolite profiles at harvest (**Figure 5A**), revealing a strong variation between cultivars regardless of the location and season, with the exception of cv. Tempranillo, which was unequivocally affected by the location factor. The correlation values were used as distance coefficients to build an HCL dendrogram, which described the berry metabolome in greater depth (**Figure 5B**). Still, the HCL dendrogram showed significant varietal influence, whereas the vineyard location and season had no significant impact (**Figure 5B**).

Principal component analysis was subsequently used to unravel the location and season effects specifically on Tempranillo berries (**Figure 5C**), and the result revealed a strong sample separation according to location. In addition, Tempranillo samples at each location grouped according to the seasons (**Figure 5C**). When correlation-based network analysis was performed on Tempranillo harvest data (**Figures 5D, E**), the results showed that major anthocyanins at MR were negatively correlated with cumulative minimum temperature (Relx) (**Figure 5D**). In RN network, only a few flavan-3-ols (procyanidin B1, B3), flavonols (kaempferol-3-glucoside, kaempferol-3-glucuronide), and astilbin were negatively correlated with Relx (**Figure 5E**). Interestingly, most of the flavonols and flavanonols at the warmer RN were positively correlated with DDD (**Figure 5E**). Of all cultivars, Tempranillo seemed to be the most location- and season-sensitive cultivar; and cumulative minimum temperature the most influencing environmental factor.

**Figure 6:**
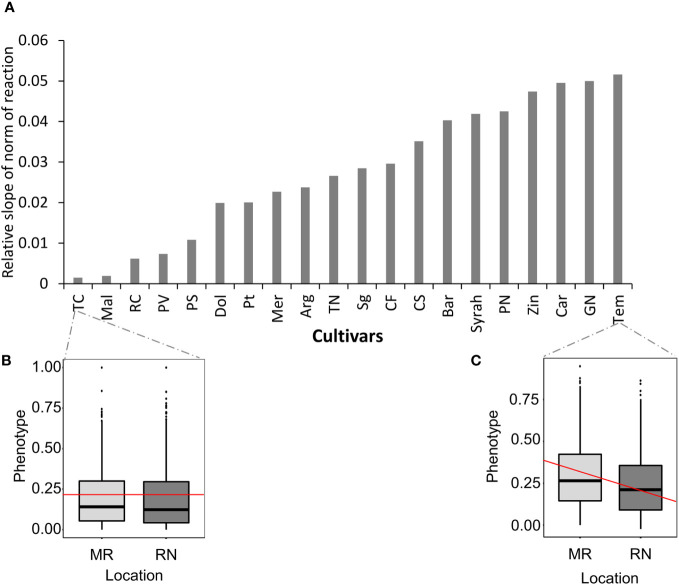
Norm of reaction. **(A)** Bar graph of the absolute slope value computed for all cultivars. **(B, C)** Box plots of selected cultivar for Mitzpe Ramon (MR) and Ramat Negev (RN) sites; red lines represent slope. The different cultivars were chosen to demonstrate different genotypic versus location and season contributions on the observed cultivar. The mean values across all metabolites for each cultivar in each location and season were used to generate the slope for each cultivar. The norm of reaction analysis was generated using harvest data in season 2017, 2018, and 2019.

Next, we ranked cultivars according to their plasticity to measured location and seasonal temperature changes, by calculating the norm of reaction for all metabolite traits (Pigliucci, 2005). In the norm of reaction plot, the slope represents phenotypic plasticity (Toubiana et al., 2020); thus, the higher the slope, the greater the phenotypic plasticity of a particular genotype for a particular trait in response to the location and season (**Figure 6A**). The more moderate slope for Tinta Cao and Malbec indicates that the differences between MR and RN sites were mainly due to the interaction between season and location (**Figure 6B**). In contrast, Tempranillo displayed the steepest slope (**Figure 6C**). These results are based on the changes across all metabolites, however, a given cultivar may have high plasticity for one trait and low plasticity for another. For a few metabolites, the observed differences between locations were specific to the variety, while for others it was the interaction between cultivar, season and location the main factor explaining their variance (**Supplementary Figure 5A**). For example, among metabolites, rutin exhibited the steepest slope (**Supplementary Figure 5C**), indicating that the differences between MR and RN sites were largely attributed to a consistent climate variation between location across the seasons, while other metabolites such as malvidin 3’’6’’-caffeoyl-glucoside) and malvindin‐3‐O‐(6″‐acetyl‐glucoside) displayed more moderate slopes, (**Supplementary Figure 5B**), suggesting that differences between MR and RN sites were due to the contributions of cultivar and season.

### The differences in gene expression profile between locations were developmental and varietal dependent

**Figure 7:**
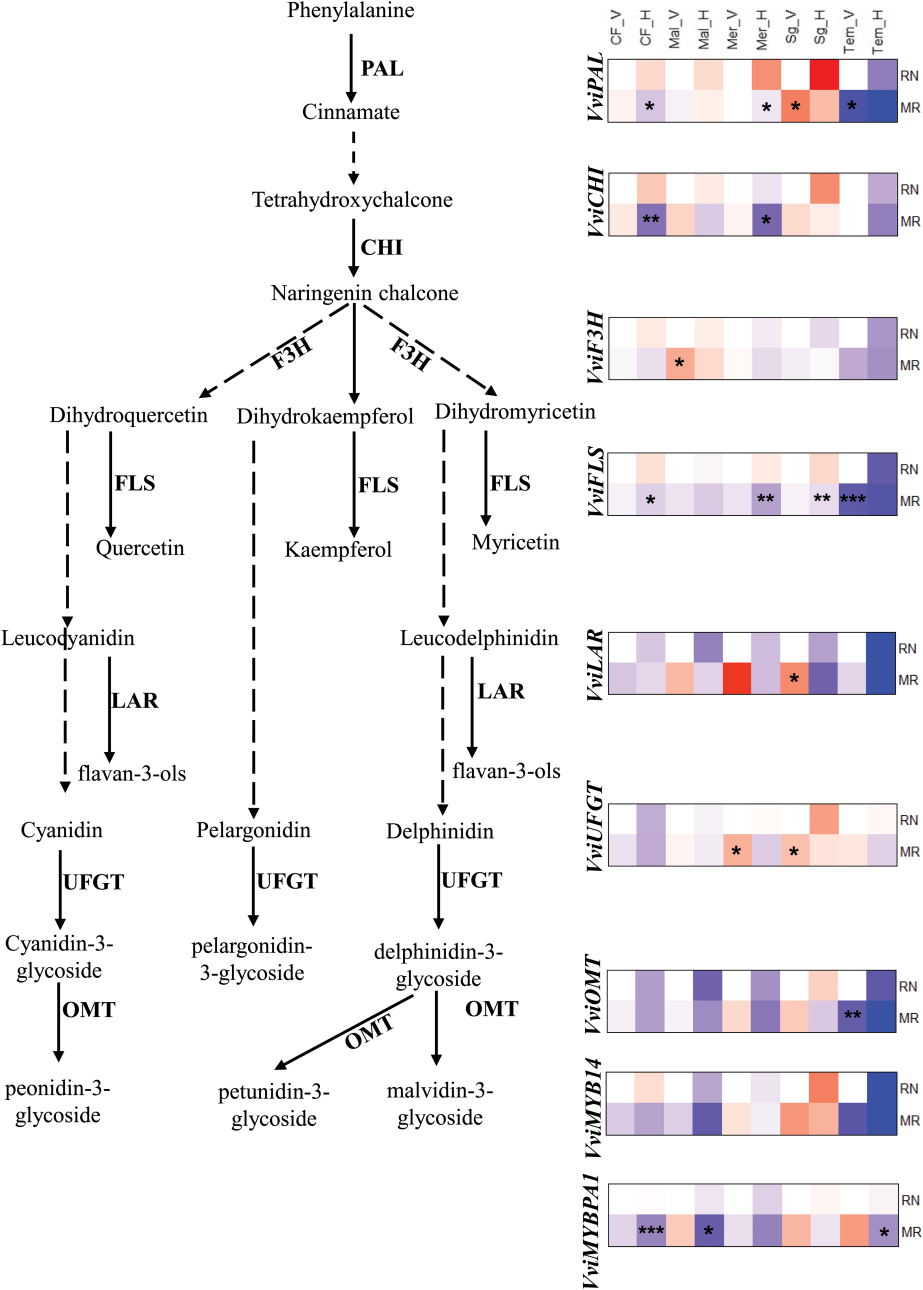
The expression profiles of phenylpropanoid pathway genes in *véraison* and fully matured berries (H) of Cabernet franc (CF), Malbec (Mal), Merlot (Mer), Sangiovese (sg) and Tempranillo (Tem) wine grape varieties grown in Mitzpe Ramon (MR) and Ramat Negev (RN) in season 2019. Gene expression visualized by heatmap as relative log2 fold change using RN *véraison* (RN_V) sample of corresponding variety as a control. Red block indicates higher gene expression while blue block indicates lower gene expression in berry skin samples collected from two locations at two different stages. Statistical significance was analysed by the Kruskal-Wallis rank sum test, followed by Dunn’s (1964) Kruskal-Wallis multiple comparisons. Asterisks indicate the significant change in MR sample in compared to RN samples (* P< 0.05; **, 0.01; ***, 0.001). PAL, phenylalanine ammonia‐lyase; CHI, Chalcone-flavonone isomerase, F3H, flavanone 3-hydroxylase; FLS, flavonol synthase; LAR, leucoanthocyanidin reductase; UFGT; UDPG-flavonoid-3-O-glucosyltransferase: OMT, O-methyltransferase.

To examine if the observed differences between sites in phenylpropanoid metabolites are related to the expression of key genes in the phenylpropanoid metabolism, we performed targeted gene expression analysis on the berry skin of five cultivars: Cabernet franc, Merlot, Malbec, Sangiovese, and Tempranillo. These cultivars were selected for their phenological and metabolic responses to environmental conditions. Tempranillo was chosen for its sensitivity to climate, Sangiovese for its consistent metabolic response to locations and seasons, and Cabernet Franc, Merlot and Malbec for their significant inter-seasonal phenological variation at the warmer RN vineyard (Gashu et al., 2020). The results showed that the effect of environmental conditions at the gene expression levels was developmental and varietal dependent. For example, comparing varieties, the transcript levels of both the upstream and downstream phenylpropanoid pathways were more strongly downregulated in Tempranillo than in other varieties at both sites, particularly during harvest (**Figure 7**). The reduction in transcript levels in Tempranillo berries from *véraison* to harvest was higher at the warmer RN site than at the cooler MR site, suggesting that this could be a reason for the lower flavonol and anthocyanin accumulation at RN compared to MR. However, there was not always a direct link between the gene expression and the metabolic data for other varieties, probably due to the involvement of non-linear reaction kinetics mechanisms, enzyme activity, substrate affinity, and post-translational modifications (Zelezniak et al., 2014; Chu et al., 2019).

The genes involved in flavonol biosynthesis, such as *VviF3H*, *VviFLS and VviLAR* were expressed at similar levels between locations in *véraison* berries, with the exception of Tempranillo in which *VviFLS* gene was significantly reduced at MR. In contrast, in fully matured berries the expression of *VviFLS* was significantly reduced in Cabernet Franc, Merlot and Sangiovese at MR compared to RN, indicating that the differences between sites in gene expression were developmental and varietal dependent. The transcript levels of UDP-glucose:flavonoid 3-O-glucosyltransferase *(VviUFGT)*, anthocyanin-O-methyltransferase *(VviOMT)* and the transcription factor MYB (*Vvi*MYB14) were generally down regulated at harvest without clear differences between sites. An exception to this was *Vvi*MYBPA1 which was strongly downregulated at cooler MR site. Our results are supported by prior works that show the UFGT gene expression increase observed in the initial 7 days after *véraison* under low temperature, which remains consistent in both high and low temperature treatments 21 days post *véraison* (Mori et al., 2007; Movahed et al., 2016).

The mechanism behind the suppression of anthocyanin accumulation in berry skins during high-temperature ripening conditions remains unclear. In certain red-pigmented plants, such as apples, the less intense red color observed in fruits ripening at higher temperatures has been linked to the downregulation of genes associated with anthocyanin biosynthesis (Lin-Wang et al., 2011). However, such an effect was not observed in most of the wine grape varieties including Cabernet Sauvignon berries ripening under control and higher-temperature conditions, suggesting that anthocyanin levels may be reduced in ripening berry skins by chemical and/or enzymatic degradation (Mori et al., 2007). Hence, the slower rate of anthocyanin accumulation in berries exposed to high temperatures after *véraison* is more likely attributed to the direct inhibition of enzymes within the phenylpropanoid biosynthesis pathway rather than the synthesis of the corresponding mRNAs.

## Discussion

Elevated temperature has already challenged many wine-producing regions. However, it is hypothesized that anticipated impacts of climate change on viticulture industry could be mitigated by exploiting existing grapevine diversity (Wolkovich et al., 2018; Biasi et al., 2019; Antolín et al., 2020; Frioni et al., 2020). In the present study, we show that polyphenol compounds in berries are strongly influenced by cultivar, location, and season and show what could be regarded as a deregulation of key genes in the pathway. In a previous study, we showed that red cultivars had a later and longer ripening phase than white ones, increasing the risk of exposing clusters to summer heat, some of which even failed to reach adequate quality standards of ripening (Gashu et al., 2020). In the current study, considerable differences among cultivars and seasons were observed; however, the warmer site was generally coupled with lower polyphenol content, particularly flavonols and anthocyanins in both *véraison* and fully matured berries. This reduction was not related to the expression levels of key genes related to phenylpropanoid metabolism. The decrease in major flavonols and anthocyanins at the warmer RN site is likely due to longer exposure of berries to heat stress, which is generally a cause of flavonol and anthocyanin degradation and repression of synthesis in berries (Zarrouk et al., 2016). Berries could also be vulnerable to dehydration under high VPD conditions (Zhang and Keller, 2015), potentially leading to adverse effects on chemical biosynthesis and accumulation within the berries. The decrease in anthocyanin content observed at the warmer RN site may also be attributed to a reduced hydroxylation process within the anthocyanin biosynthetic pathway (Tarara et al., 2008). Increased gene expression levels at the warmer site might be a compensatory means of the berry. Evidence already exists showing a poor association between phenylpropanoid content in the berry skin and corresponding gene transcripts under elevated temperatures. For example, studies in Cabernet Sauvignon berry showed a greater reduction of total anthocyanin content under high temperature (35°C) due to anthocyanin degradation, which was not related to the downregulation of anthocyanin biosynthetic genes (Mori et al., 2007).

Lower flavonol and anthocyanins contents in ripe berries at RN are presumably due to a higher pace in reduction from *véraison* to harvest. The analysis of the norm of reaction revealed that the observed variance in metabolite content was metabolite- and variety-specific or due to the interaction between cultivar, location, and season. Among phenolic compounds, the most inconsistent response to environmental conditions was observed in the stilbenes (*Cis-* piceid, *trans*-piceid and D-viniferin), which increased at the warmer RN site in 2019 but not in the 2017 and 2018 seasons. A possible explanation for these differences in the polyphenol metabolism might be the fact that stilbenes are generally associated with biotic stresses and relatively less with abiotic cues (Chong et al., 2009).

The results from the varietal collection shown here are generally in support of other investigations showing that heat treatment during ripening reduced flavonol and anthocyanin concentrations in potted Sangiovese and Merlot berries (Pastore et al., 2017; Yan et al., 2020). In another study, high temperatures (28/18°C day/night) increased flavonol accumulation (Torres et al., 2017). In a third study, a combination of elevated temperature and CO_2_ treatment led to an increase in anthocyanin concentration (Martínez-Lüscher et al., 2016) in Tempranillo berries (Martínez-Lüscher et al., 2016). This inconsistency among studies emphasizes the complexity of the nexus between the grape variety and environmental elements, far from being entirely unravelled. Notably, in the current study, under the field conditions used, we showed great varietal diversity in response to environmental conditions. The correlation based HCL dendrogram, which described the berry metabolome in greater detail, revealed major differences between the cultivars, less than those between locations or season except for Tempranillo, for which flavonols and anthocyanins contents were consistently lower at the warmer RN than the cooler MR. This is consistent with its phenology as described previously by Gashu et al. (2020); Tempranillo was the most hyper-sensitive cultivar, consistently displayed the largest phenological shift between sites, as opposed to Petit Verdot, Malbec, Cabernet Franc, Cabernet Sauvignon, and Petit Syrah, which displayed relatively small phenological shift.

Network analysis of the metabolite data revealed the differences between locations within the relation between metabolites. The RN metabolite network was characterized by increased number of edges, indicating the readiness of metabolic rewiring in response to environmental stress as described earlier (Hochberg et al., 2013). This has also been shown in a co-expression network analysis of metabolic genes from publicly available expression data of *Arabidopsis thaliana* plants exposed to heat and drought stress (Fait et al., 2020). Taken together, our analysis supports the notion that stress conditions lead to metabolic network tightening, likely resulting in improved concerted regulation under sub-optimal conditions. Furthermore, the positive correlations between flavonols and anthocyanins in the RN network, and negative correlation in the MR network suggest a different regulation of carbon allocation from phenylalanine towards anthocyanins as a result of heat stress. In both networks, when integrating environmental data, temperature and radiation were only loosely linked to metabolites, which is like a consequence of the differential response of individual cultivars to the environmental elements measured. When the network analysis was performed for each cultivar, metabolites did show a strong correlation with radiation, Hs, Relx, and DDD. Our results emphasize the relevance of grapevine genetic diversity and varietal choice in future viticulture when considering the implication of global warming on grape growing areas.

## Conclusion

This detailed three-year study on phenylpropanoid metabolism and regulation in *Vitis vinifera* red cultivars indicates that the genetic diversity of grapevine is key in adapting viticulture to warmer climates. Under the factors tested and characterized here, both season and location were found to be predominant factors affecting berry metabolism. We showed considerable reduction of polyphenol contents, particularly flavonols and anthocyanins at the warmer site, the severity of which depends on cultivar and season. Our findings highlight the importance of careful cultivar selection for future high-quality grape production, particularly in regions already suffering from heat stress. Future studies should improve our understanding on the regulation of polyphenol gene expression under stress conditions and its link with processes like anthocyanin degradation.

## Data availability statement

The raw data supporting the conclusions of this article will be made available by the authors, without undue reservation.

## Author contributions

AB and AF conceived and planned the study. KG, TA and AB collected the berry samples in the field. PV did the gene expression analysis. KG prepared the berry samples for extraction, performed the sample extraction and data analysis and analysis using the UPLC-MS device, and wrote the body of the manuscript with AF. All authors contributed to the article and approved the submitted version.

## Glossary

**Table:**

Delphinidin‐3‐glu-coum	Delphinidin‐3‐O‐(6′′‐p‐coumaroyl‐glucoside)
Petunidin‐3glu‐coum	Petunidin‐3‐O‐(6′′‐p‐coumaroyl‐glucoside)
Pet‐3‐glu-Acet	Petundin‐3‐O‐(6′′‐acetyl‐glucoside)
	
Cyan‐3‐glu-Acet	Cyanidin‐3‐O‐(6′′‐acetyl‐glucoside)
Cyan‐3‐glu-coum	Cyan‐3‐glu, Cyanidin‐3‐O‐glucoside;Delph‐3‐glu-Acet, Delphinidin‐3‐O‐(6′′‐acetyl‐glucoside)
Delph‐3‐glu	Delphindin‐3‐O‐glucoside
Kaemp‐3‐glr	Kaempferol‐3‐O‐glucuronide
Kaemp‐3‐glu	Kaempferol‐3‐O‐glucoside
Mal‐3‐glu-Acet	Malvindin‐3‐O‐(6′′‐acetyl‐glucoside)
Malvidin‐3‐glu-caffeoyl	Malvidin‐3‐O‐(6′′‐caffeoyl‐glucoside)
Malvidin‐3‐glu-coum	Malvidin‐3‐O‐(6′′‐p‐coumaroyl‐glucoside)
Malvidin‐3‐glu	Malvidin‐3‐O‐glucoside
Myricetin‐3‐glr	Myricetin‐3‐O‐glucuronide
Myricetin‐3‐glu	Myricetin‐3‐O‐glucoside
Isorhamentin-3-glu	Isorhamentin‐3‐O‐glucoside
Naringenin ch‐4-glu	Naringenin‐chalcone‐4‐O‐glucoside
Peonidin‐3‐glu-Acet	Peonidin‐3‐O‐(6′′‐acetyl‐glucoside)
Peo‐3‐glu-coum	Peonidin‐3‐O‐(6′′‐p‐coumaroyl‐glucoside)
Peonidin‐3‐glu	Peonidin‐3‐O‐glucoside
Petunidin‐3‐glu	Petunidin‐3‐O‐glucoside
Quercetin‐3‐glr	Quercetin‐3‐O‐glucuronide
Quercetin‐3‐glu	Quercetin‐3‐O‐glucoside
Rutin	Quercetin‐3‐O‐rutinoside
	
MR	Mitzpe Ramon
RN	Ramat Negev
