# Supplementary material for: Temperature differences between sites lead to altered phenylpropanoid metabolism in a varietal dependent manner
Source: Front Plant Sci. 2023 Oct 19;14:1239852. doi: 10.3389/fpls.2023.1239852 (PMC10620969; doi:10.3389/fpls.2023.1239852)
Supplement: Supplementary file 1 [file DataSheet_1.pdf]

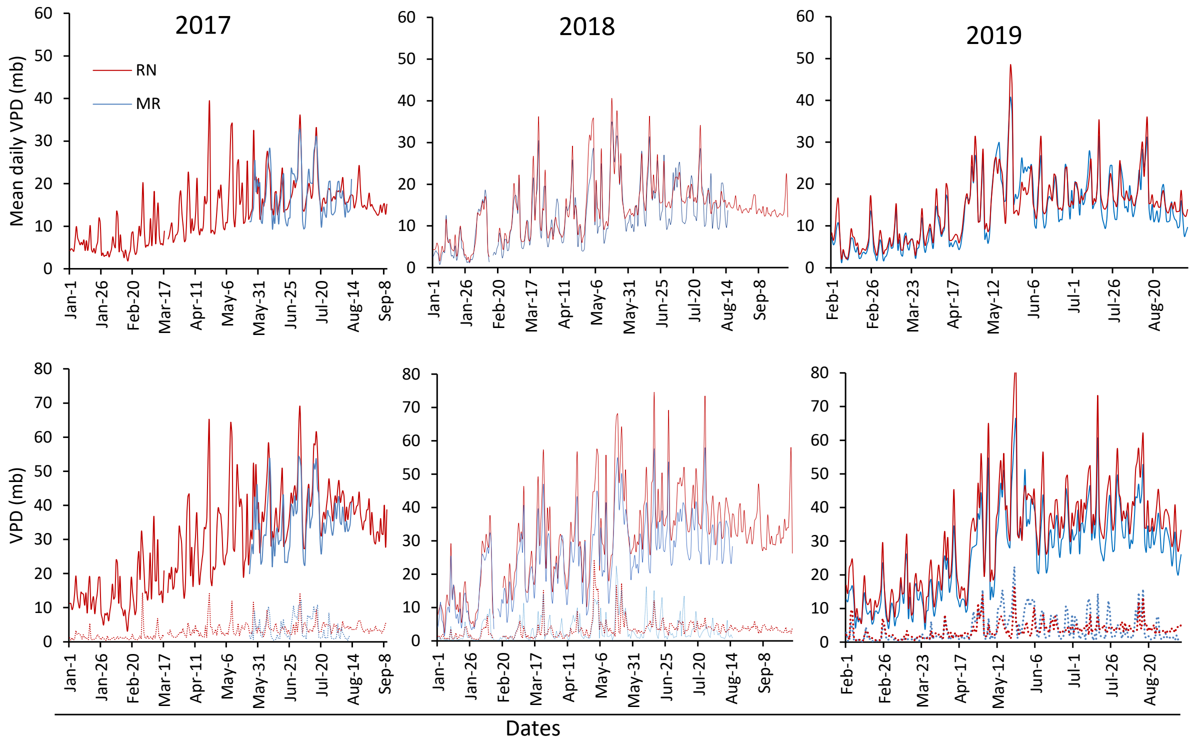

Supplementary Figure 1. Vapor pressure deficit (VPD) at the experimental sites during 2017–2019 seasons. The solid and dotted lines denote maximum and minimum measurements, respectively. RN, Ramat Negev; MR, Mitzpe Ramon. The 2017 data at Mitzpe Ramon vineyard are from 23rd May 2017 until harvest end.

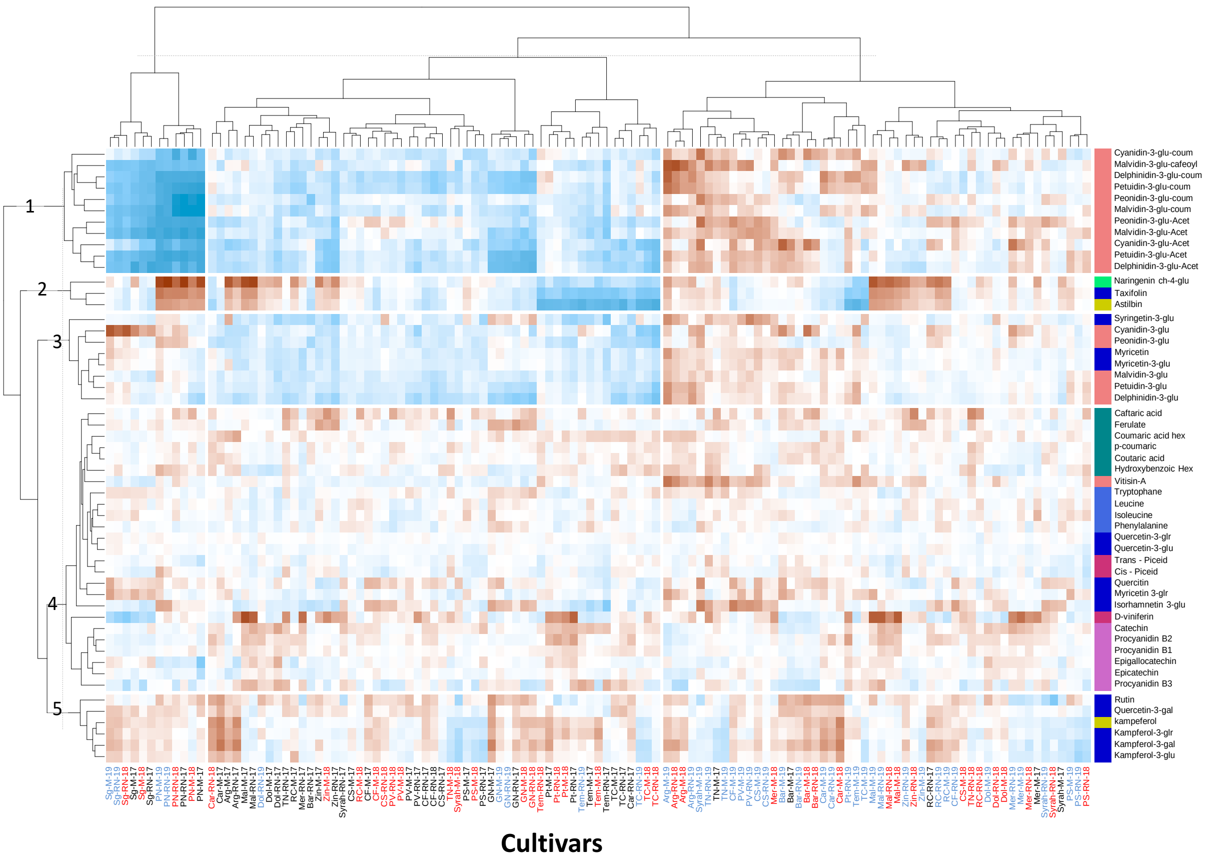

Supplementary Figure 2: *Heatmap of skin polyphenols in red grapevine berries at véraison. The heatmap was generated using the mean value of four biological replicates following normalization to the median of each metabolite on all cultivars and log2 transformation. Cultivar names are denoted by vineyard abbreviations (MR and RN) followed by vintage (17,18 or 19). Colored cultivar names indicate samples collected in 2017 (black), 2018 (red), and 2019 (blue). Red and blue rectangles represent an increase and decrease of metabolite relative to the median. MR, Mitzpe Ramon; RN, Ramat Negev*

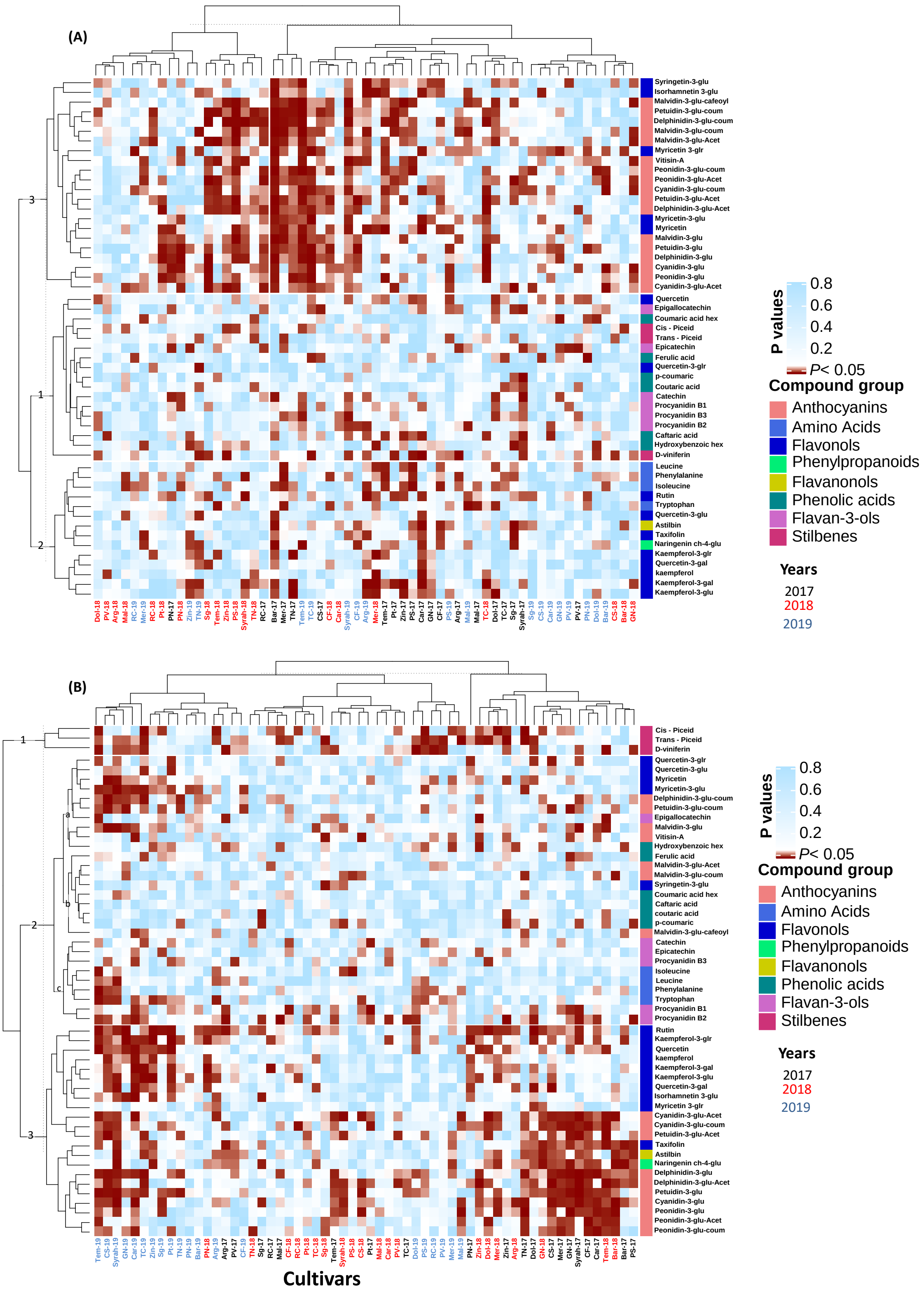

***Supplementary Figure 3**: heatmap of skin polyphenols at véraison **(A)** and harvest **(B)** that changed significantly between Mitzpe Ramon (MR) and Ramat Negev (RN) vineyards during 2017, 2018 and 2019 seasons. Heatmap representation of significant metabolites identified by nonparametric t test between MR and RN. Each metabolite was individually compared between locations for each cultivar in each season. Red coloured rectangles indicate a significant change (P<0.05) of metabolites between locations. Cultivar names are composed by vintage abbreviations (17, 18, 19). Colored cultivar names indicate samples collected in 2017 (black), 2018 (red), and 2019 (blue).*

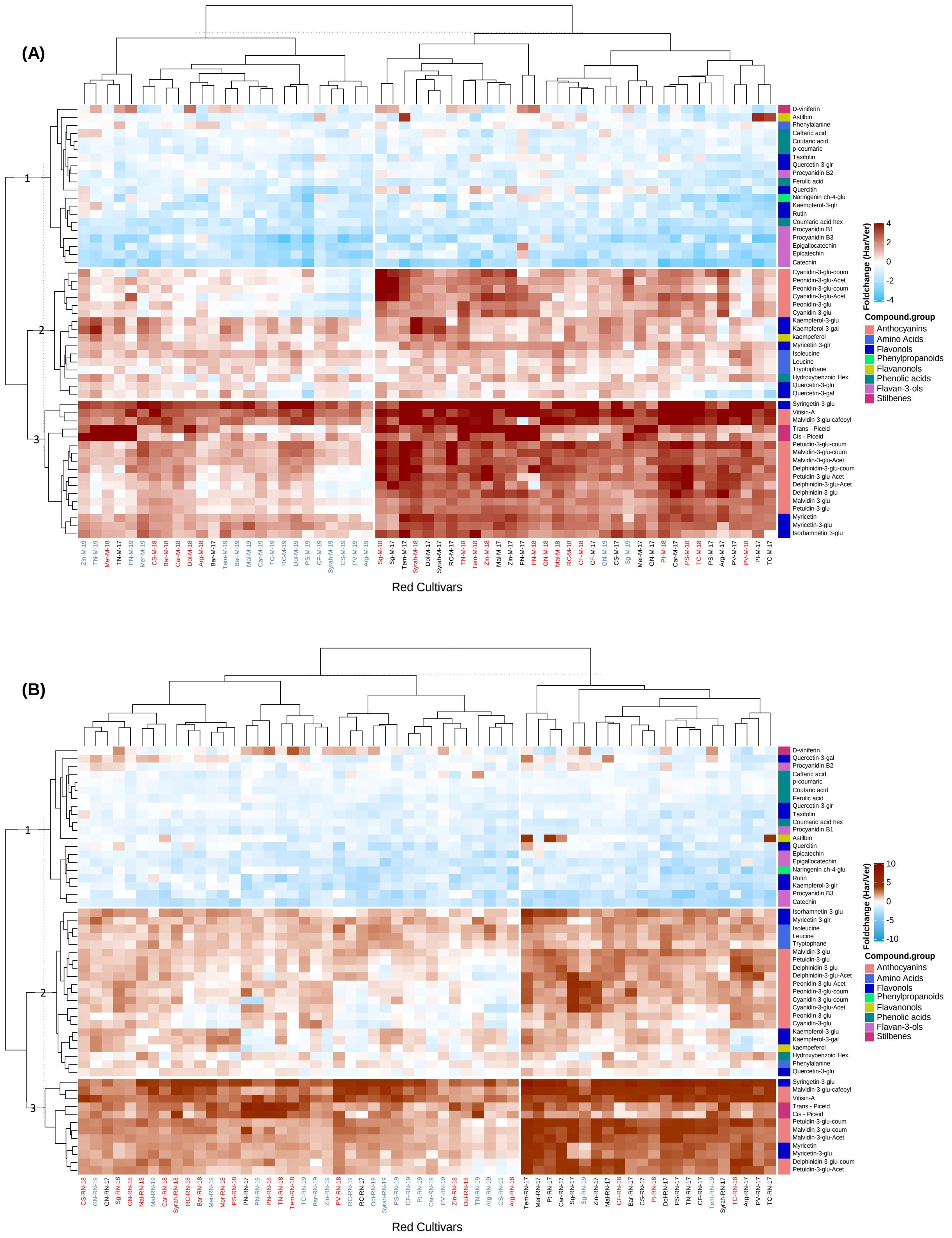

***Supplementary Figure 4**: Change in skin polyphenols from véraison (Vér) to harvest (Har) at Mitzpe Ramon **(A)** and Ramat Negev **(B)** vineyard from 2017-2019, expressed as foldchange (Har/Vér). The mean value of four biological replicates of each metabolite for each cultivar was calculated separately in each season. Then, the values at harvest were divided by the values at véraison. The hierarchical clustering heatmap was generated following log2 transformation. Cultivar names are composed by location abbreviation [M (mitzpe Ramon), RN (Ramat Negev)] and vintage (17, 18, 19). Colored cultivar names indicate samples collected in 2017 (black), 2018 (red), and 2019 (blue). Colored rectangles represent metabolite increases at harvest (red) and véraison (blue).*

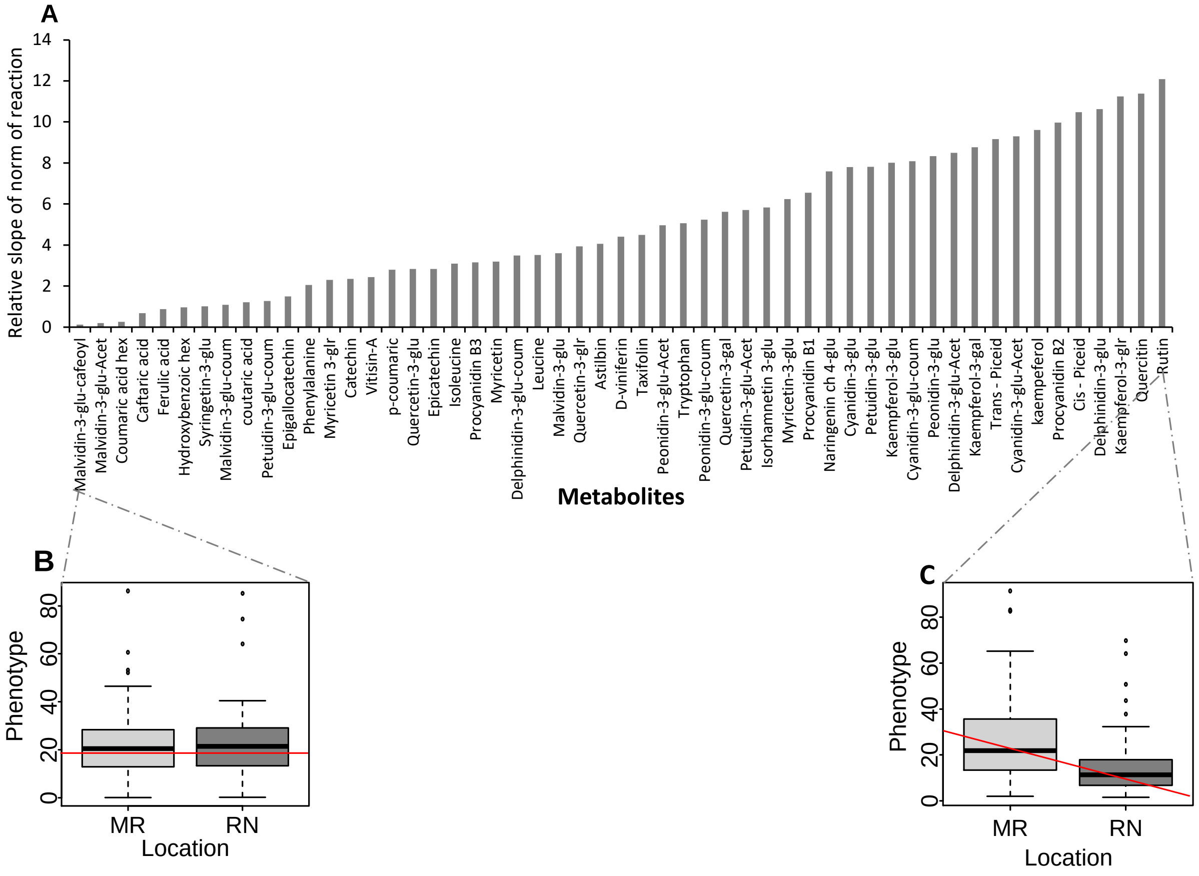

***Supplementary Figure 5**:* Norm of reaction. (A) Bar graph of the absolute slope values computed for all metabolites. (B), (C) Box plots of selected metabolites for Mitzpe Ramon (MR) and Ramat Negev (RN) sites; red lines represent slope. The different metabolites were chosen to demonstrate different genotypic versus location and season contributions to the observed cultivar. The slope for metabolites were generated using mean values of each metabolite across all cultivars in each location separately. The norm of reaction analysis was generated using harvest data in season 2017, 2018, and 2019.

Supplementary Table 1: Differences between vineyards in the anthocyanin contents in ripe red berries grown at Mitzpe Ramon (MR) and Ramat Negev (RN) vineyards during 2019 season.

**Cultivar**	**Glycosylated anthocyanins (µg g. DW^{-1})**									
	**Cyanidin-3-glu**		**Petuidin-3-glu**		**Peonidin-3-glu**		**Malvidin-3-glu**		**Delphinidin-3-glu**	
	**MR**	**RN**	**MR**	**RN**	**MR**	**RN**	**MR**	**RN**	**MR**	**RN**
PV	2.27±0.2.0	7.04±2.44	1.88±0.13	3.42±0.53	0.17±0.02	0.52±0.19	9.80±0.61	13.92±1.20	1.16±0.10	2.24±0.46
TC	2.38±0.24	2.75±0.32	1.50±0.10	1.21±0.14	0.12±0.03	0.28±0.05	7.57±0.39	8.63±0.79	0.97±0.03	0.62±0.08
Dol	3.89±0.32	4.85±0.32	1.52±0.20	1.40±0.21	0.23±0.04	0.32±0.02	7.01±0.82	7.14±0.75	1.09±0.10	0.84±0.11
Mal	3.93±0.41	12.94±2.28	2.64±0.26	3.69±0.44	0.30±0.05	1.20±0.18	10.11±0.63	11.93±0.79	1.66±0.17	2.47±0.36
CS	5.27±0.74	3.73±0.64	1.73±0.16	1.15±0.16	0.51±0.06	0.52±0.09	8.14±0.41	8.24±0.68	1.57±0.26	0.82±0.07
PS	5.47±0.98	5.02±0.56	4.31±0.40	3.76±0.25	0.37±0.05	0.38±0.05	12.44±0.55	14.35±1.23	3.29±0.40	2.37±0.23
RC	6.23±1.24	10.85±0.63	2.12±0.16	3.54±0.42	0.60±0.13	1.19±0.08	8.86±0.38	14.34±0.83	1.42±0.16	2.28±0.40
CF	6.29±0.85	8.16±1.65	1.92±0.19	2.09±0.20	0.57±0.08	0.83±0.18	7.79±0.38	9.97±0.66	1.62±0.22	1.53±0.25
Car	9.29±1.25	6.57±2.00	4.52±0.44	3.55±0.39	0.29±0.06	0.19±0.06	11.19±0.93	10.46±0.69	4.74±0.63	3.06±0.40
GN	9.86±1.94	9.86±3.06	0.85±0.07	0.54±0.13	1.11±0.17	0.86±0.33	5.97±0.52	4.62±0.71	0.53±0.06	0.27±0.05
Syrah	10.08±0.59	5.54±0.32	2.39±0.26	1.57±0.11	1.22±0.08	0.96±0.05	8.69±0.26	8.12±0.41	1.59±0.31	0.66±0.01
Pt	10.22±0.64	9.27±2.29	4.19±0.31	4.06±0.37	0.36±0.01	0.46±0.13	11.79±0.69	13.88±1.41	4.35±0.49	3.86±0.63
TN	10.59±1.69	11.89±1.75	1.96±0.17	1.93±0.17	1.47±0.24	1.76±0.21	9.49±0.66	11.38±0.85	1.29±0.20	0.94±0.12
Mer	10.59±1.31	18.82±3.89	2.64±0.24	3.36±0.34	0.51±0.07	1.18±0.30	8.85±0.52	11.59±0.53	2.60±0.23	2.75±0.26
Zin	11.35±2.54	10.29±1.71	2.21±0.40	2.30±0.42	0.90±0.20	0.78±0.10	9.86±0.73	10.45±1.32	1.42±0.28	1.26±0.26
Arg	11.62±0.65	9.49±2.85	**5.68±0.56**	**5.61±0.91**	0.88±0.06	0.88±0.26	**14.49±1.36**	**18.42±2.25**	4.89±0.66	4.25±0.99
PN	19.97±1.32	31.76±4.08	2.24±0.14	2.80±0.11	**3.64±0.24**	**4.84±0.27**	11.03±0.74	13.12±0.54	1.23±0.06	1.49±0.09
Bar	22.93±1.82	20.14±6.42	3.87±0.11	3.09±0.70	0.32±0.03	0.75±0.50	9.04±0.23	8.45±1.17	2.99±0.12	2.27±0.66
Tem	23.28±0.42	13.91±2.60	4.21±0.33	2.85±0.34	0.92±0.05	0.62±0.10	9.60±0.55	8.06±0.66	4.73±0.26	2.49±0.43
Sg	**157.66±10.5**	**142.45±14.75**	3.88±0.26	4.06±0.47	2.63±0.13	2.28±0.22	7.47±0.53	8.02±0.64	3.21±0.36	3.34±0.48

Error bars are standard error (*n*=4). *Means with bold indicate significant differences between locations (P < 0.05) within the same cultivar based on a non-parametric t-test.*

Supplementary Table 2: Summary of Mitzpe Ramon and Ramat Negev correlation networks that were constructed using UPLC-QTOF-MS generated metabolite profiles from the skin of ripe red grapevine berries and climate indices during ripening.

Network summary	Ramat Negev	Ramon
Number of nodes	56	53
Number of edges	222	216
Average number of neighbours	7.929	8.151
network diameter	6	6
network radius	4	3
characteristics path length	2.167	2.485
clustering coefficient	0.495	0.507
network density	0.205	0.157
network heterogeneity	0.567	0.521
network centralization	0.248	0.137
